# Operation for Strangulated Hernia; Removal of Hernial Sac, Followed by Radical Cure

**Published:** 1863-06

**Authors:** J. F. Miner


ART. IV.—*Operation for Strangulated Hernia; removal of hernial sac, followed by radical cure.* BY J. F. MINER, M. D.

March 17, 1863, was called to visit Mr. J. Bumm, by his medical attendant, Dr. L. Krombein, who had failed to reduce a strangulated oblique inguinal hernia, after protracted but very judicious effort. The strangulation had been of three days standing, but the symptoms were not severe, the protrusion consisting mostly of omentum. The hernia was old, and had been neglected; the patient had not worn a truss, though he had suffered from rupture for ten or twelve years. Patient was 62 years old, and usually vigorous. Dr. Krombein being professionally engaged Dr. J.

R. Lothrop was kind enough to visit the patient and assist in the operation. After obtaining the full effects of sulphuric ether, the attempt was made to reduce the hernia, but failing in this, the usual operation for hernia was made. On opening down upon the sac, it was found to be large and unusually thickened; the walls were from one-half to three-quarters of an inch in thickness, dense and firm. The inner surface was red and granular, and no difficulty presented in obtaining reduction of the intestinal protrusion, but when this was accomplished the sac was yet present, forming a tumor the size of a man's closed hand, so firm and unyielding as indeed to suffer no apparent reduction in size after evacuation of its contents.—The immense thickness of the walls of the sac had necessitated a quite lengthy incision before reaching its interior, which had been made by most careful dissection.

The question at once suggested itself, what shall be done to get rid of this tumor and to prevent a return of the contents of the sac? The contraction of the walls of the protruded peritoneum, usual after operation for hernia could not in this case take place; the cavity remained open and the thickened walls unyielding. The sac had what may be called a a body, and a neck. The body was large and broad, at least three inches in diameter, while the neck was much smaller, perhaps one inch and a half in diameter. After reflecting a moment that the incision into the peritoneum would not be greater by making amputation of the neck than had already been made through the body of the tumor, and that by this means the walls might by approximated so as possibly to radically close the opening; and also remembering that puncture, into the peritoneal cavity, was as frequently followed by acute peritonitis, as more free incisions; with the consent of my assistant I divided the neck of the peritoneal tumor, separated it carefully from the spermatic cord and testicle and removed it entire.

It had formed extensive attachments to the surrounding parts and a few small arteries were ligated. After all hemorrhage had ceased, sutures were introduced not only through the skin, but deeply, so that in closing the external wound the divided walls of the sac would be brought in contact with each other. Water dressings were applied and full anodyne directed.

This patient recovered without unpleasant symptoms of any kind; the scrotum was largely swollen and infiltrated with serum, but there was nothing to retard the rapid recovery of the patient. The result so far, was highly satisfactory, and when we find that he not only made rapid recovery from the operation, but that he had also obtained a radical cure of his hernia, it is something more than satisfactory, it is an achievement, which I take pride in relating; not so much for myself as the art I am allowed to practice. Four weeks from the time of operation on visiting my former patient, I find him wheeling dirt in the garden, without *truss*, which I had advised him to wear as a protection, and upon my expressing a fear that he would re-produce the rupture, he commenced to cough violently, and assure me that it was all right. Being German he could show, better than tell it, and made immediate exhibition of the parts, demonstrating the complete and perfect cure of his hernia.

Excision of the hernial sac, was long ago practiced for the radical cure of hernia, but as a common operation for the radical cure of reducable hernia it would not now be suggested. It is quite possible that surgeons have met similar cases to the one above described, and even treated them in similar manner, but record of the fact is not easily obtained. While no surgeon would now suggest excision of the sac for radical cure of reducible hernia, still if strangulation had taken place, and an operation made necessary, we can hardly appreciate any greatly increased risk from removal of such a tumor by excision over opening it, relieving the stricture and allowing the mouth of the sac to remain unclosed, open again to receive the hernial protrusion. This case proves nothing so far as establishing the feasibility of such operation, though the result justifies the procedure in this instance. At a remote period of the profession "in scrotal hernia, the testicles were often extirpated along with the hernial sac: and so common had this practice become in the seventeenth century, that, as Dionis informs us, an itinerant operator was in the habit of feeding his dogs with the organs which he had thus removed." (Gross's Surgery.)

The most approved operations in surgery, if practiced thus barborously, would soon fall in disrepute; yet it is by no means intended to advocate excision of the hernial sac, for radical cure of reducible hernia, or even to express the opinion that excision of the sac would ordinarily be justifiable even if strangulation had taken place, and an operation for relief of the stricture made necessary. We are influenced often by success, and led to incorrect views and improper practice by the results of a limited observation, when more prolonged experience will show the error and danger of our course. In reflecting upon this operation and estimating as accurately as possible the dangers and advantages which it offers, the conclusion is almost unavoidable that in a case similar to the one described, excision of the sac cannot greatly, if at all, increase the danger of peritonitis, or in any way augment the risk of life. In making it a great many times, there can be no doubt of its frequent failure in effecting a radical cure, and of the operation being often followed by fatal terminations; this is true of all operations for hernia. That it would more frequently result in cure of the rupture than if treated in the usual manner, would seem probable; that it would be attended by greater danger to life is by no means certain, indeed there is no ground for belief that such would be the result.

It will be recollected that the hernial sac had, in the case described, become greatly thickened, and the structure and character of the peritoneal membrane wholly changed. It is highly probable that cutting, puncturing or excising such a morbid product as this, is much less liable to be attended by inflammation dangerous to life than removal of a recently protruded portion of peritoneal membrane. This structure partakes much more of the character of false membrane when great thickening has been produced, than of the serous membranes, and is not like them so liable to take on violent inflammatory action upon slight provocation. This is a point of great importance, and should be duly considered in connection with the dangers of peritonitis which are known to be present after wounds of all kinds of this membrane.

That this operation was a justifiable and desirable one in this instance, we have no doubt; that it would be proper in most similar cases we have no hesitation in believing; but we hope that no one will misunderstand the statements made, and conceive that a new operation is proposed, or an old one revised and advocated.

